# Step by Step: Evaluation of Cardiorespiratory Fitness in Healthy Children, Young Adults, and Patients with Congenital Heart Disease Using a Simple Standardized Stair Climbing Test

**DOI:** 10.3390/children11020236

**Published:** 2024-02-12

**Authors:** Maurice Pablo Mall, Johanna Wander, Anne Lentz, André Jakob, Felix Sebastian Oberhoffer, Guido Mandilaras, Nikolaus Alexander Haas, Simone Katrin Dold

**Affiliations:** Department of Pediatric Cardiology and Pediatric Intensive Care, University Hospital, LMU Munich, 81377 Munich, Germany; m.mall@campus.lmu.de (M.P.M.); simone.dold@med.uni-muenchen.de (S.K.D.)

**Keywords:** stair climbing test, cardiorespiratory fitness, exercise test, children, congenital heart disease

## Abstract

(1) Background: Cardiorespiratory fitness (CRF) is known to be a prognostic factor regarding long-term morbidity and mortality. This study aimed to develop a standardized Stair Climbing Test (SCT) with a reliable correlation to spiroergometry and the 6MWT which can be used in healthy children as well as patients with congenital heart disease (CHD) and a restricted exercise capacity. (2) Methods: A total of 28 healthy participants aged 10–18 years were included. We tested the individuals’ CRF by cardiopulmonary exercise testing (CPET) on a treadmill, the 6MWT, and a newly developed Stair Climbing Test (SCT). For the SCT, we defined a standardized SCT protocol with a total height of 13.14 m to achieve maximal exercise effects while recording time and vital parameters. To compare the SCT, the 6 Min Walking Test, and CPET, we introduced an SCT-Index that included patient data (weight, height) and time. To assess the SCT’s feasibility for clinical practice, we also tested our protocol with five adolescents with complex congenital heart disease (i.e., Fontan circulation). (3) Results: A strong correlation was observed between SCT-Index and O_2_ pulse (r = 0.921; *p* < 0.001). In addition, when comparing the time achieved during SCT (tSCT) with VO_2_max (mL/min/kg) and VO_2_max (mL/min), strong correlations were found (r = −0.672; *p* < 0.001 and r = −0.764; *p* < 0.001). Finally, we determined a very strong correlation between SCT-Index and VO_2_max (mL/min) (r = 0.927; *p* = <0.001). When comparing the 6MWD to tSCT, there was a moderate correlation (r = −0.544; *p* = 0.003). It appears to be feasible in patients with Fontan circulation. (4) Conclusions: We were able to demonstrate that there is a significant correlation between our standardized SCT and treadmill CPET. Therefore, we can say that the SCT can be used as an easy supplement to CPET and in certain contexts, it can also be used as a screening tool when CPET is not available. The advantages would be that the SCT is a simple, quick, cost-effective, and reliable standardized (sub)maximal exercise test to evaluate CRF in healthy children on a routine basis. We can even assume that it can be used in patients with congenital heart disease.

## 1. Introduction

Physical activity offers fundamental health benefits for children and adolescents. Research has consistently shown that individuals with high cardiorespiratory fitness (CRF) present with obesity significantly less often [1,2,3], suggesting a link between fitness and body composition. Furthermore, generally elevated CRF levels are inversely related to a more favorable cardiovascular profile, indicating a protective role for cardiovascular health [1]. These effects extend even further, showing an association between increased CRF levels and a lower risk of all-cause mortality and reduced susceptibility to coronary heart disease and cardiovascular events [4,5]. These results underscore the overall benefits of physical fitness and highlight its potential to reduce risks across multiple health domains, including cardiovascular, metabolic, and psychiatric outcomes [2,6]. This is one of the reasons why the World Health Organization (WHO) recommends that children and young people aged 5–17 years should do at least 60 min of moderate to vigorous aerobic activity per day throughout the week, along with three days a week of intense aerobic activities and movements that strengthen the muscles and bones [7].

With this knowledge in mind, it is even more important for children and young adults with congenital heart disease to assess cardiorespiratory fitness on a routine basis and to maintain and/or increase the fitness level in these individuals.

To measure cardiorespiratory fitness, there are basically two types of exercise tests: maximal exercise tests and submaximal exercise tests. Maximal exercise tests require maximal effort, whereas submaximal exercise tests, on the other hand, do not require maximal effort. Cardiorespiratory fitness is therefore often estimated using equations or nomograms, leading to possible inaccuracies. Nevertheless, submaximal testing is used when maximal testing is not practical for safety, physiological, logistical, or cost reasons, and it is useful for identifying individuals with low cardiorespiratory fitness [8]. An example of a maximal exercise test is CPET on a bicycle or treadmill; the 6 Min Walking Test (6MWT) is an example of a submaximal test [8,9,10].

In Europe, bicycle ergometry is the preferred choice over treadmill ergometry due to its ability to minimize artifacts in stress ECG and alleviate the potential insecurity or anxiety caused by the moving surface of a treadmill. However, this preference comes with a trade-off, as bicycle ergometry activates less muscle mass, leading to a maximal performance level approximately 10% lower than that achievable on a treadmill. The treadmill also offers the advantage of achieving a higher heart rate compared to the bicycle [6,11].

The 6MWT is a standardized, simple, cheap, submaximal exercise test that can be carried out almost anywhere without great effort. It measures the maximum distance a subject can walk within 6 min [8,12].

In the literature, Stair Climbing Tests (SCTs) are described in many ways, with the majority of studies assessing functional mobility in patients after orthopedic surgery or with neuromuscular diseases, rather than those with congenital heart disease. However, there is currently no standardized and thereby comparable protocol for SCTs. Therefore, our working group developed a standardized SCT protocol. Our goal was to develop a simple, standardized test which is applicable in patients with congenital heart disease. Therefore, we studied the approaches described in the literature and aimed for a protocol that would induce maximal exertion in order for it to be comparable to CPET results. We had already tested it in healthy normal-weight and obese 18–30-year-old adult subjects. The results showed a significant correlation to the 6MWT and spiroergometry [13].

The aim of this study was to evaluate CRF in healthy children and adolescents aged 10–18 years using the SCT and comparing the results with spiroergometry (maximal oxygen uptake, oxygen pulse) and the 6MWT. Further, our aim was to assess CRF in children and adolescents with complex congenital heart disease, i.e., Fontan patients.

## 2. Materials and Methods

### 2.1. Ethical Statement

The “Step by Step” study was considered ethically and legally innocuous by the ethics committee at LMU Munich, No.: 22-0029, on 22 September 2022 and was conducted in accordance with the ethical standards of the Declaration of Helsinki. Informed consent was given by all subjects.

### 2.2. Study Design and Study Population

This study was a pilot study. A total number of 28 healthy participants, aged 10–18 years, were included. To be eligible for this study, subjects had to have a BMI <25 kg/m^2^. Only children and adolescents with no known pre-existing diseases and no regular medication were included. Those with an abnormal physical examination, echocardiography, or pathological finding in the 12-lead electrocardiogram (ECG) were excluded. In addition, individuals with a respiratory exchange ratio (RER) of >1.0 [14,15,16] at maximal exercise in spiroergometry were defined as eligible. Participants who terminated the exercise before a RER >1.0 were excluded. Additional five Fontan patients were comparable regarding age and had a functioning Fontan circulation with normal saturation and a normal ventricular function without significant valvular regurgitation. For the Stair Climbing Test, the test subjects only needed to be physically capable of performing the test. There were no further exclusion criteria. The examinations were performed on one or two days in the pediatric cardiology outpatient unit of LMU Munich.

### 2.3. Assessment of Cardiorespiratory Fitness

In order to measure cardiorespiratory fitness, the study participants initially had to perform a 6MWT. Following adequate rest, the SCT was conducted according to our standardized protocol. Before CPET on the treadmill, a resting period of at least 30 min was employed to ensure sufficient recovery time. This allowed the individuals to feel subjectively recovered and attain their resting heart rate.

The 6MWT was performed according to the guidelines of the German Society for Pediatric Cardiology (DGPK), which translated the instructions from the American Thorax Society and European Respiratory Society into German [9,17]. Heart rate (HR, bpm), oxygen saturation (%), and blood pressure (mmHg) were measured immediately before and after the test. Furthermore, the 6 min walking distance (6MWD) achieved was recorded.

The Stair Climbing Test required, according to our protocol and to achieve maximal exercise levels, 4 floors and/or a staircase with a total of at least 12 m height [18,19] and a chest strap to measure the subject’s heart rate during the test. Oxygen saturation, heart rate, and blood pressure were monitored before and after the test. In our study, a height of 13.14 m was used, which is the equivalent of 4 floors. The subjects had to run up and down these 4 floors as fast as possible, without gripping the handrail or skipping any steps. During the SCT, the children and adolescents were encouraged and motivated by the person conducting the test so they would achieve maximum performance. There was no upper time limit. In addition, an SCT index was calculated, which was defined as SCT-Index = ($\frac{\mathrm{body}\;\mathrm{weight}\;\times \;\mathrm{staircase}\;\mathrm{height}}{\mathrm{tSCT}}$).

The cardiopulmonary exercise test (CPET) was performed on a treadmill according to the recommendations of the DGPK using a step-protocol [10]. The speed and incline of the treadmill were increased every 90 s until the subjects were exhausted. The speed started at 2.5 km/h and was increased by 0.5 km/h every 90 s; the incline started at 0% and was increased by 3% every 90 s up to a maximum incline of 21%. After that, only the speed was increased [10]. Different CPET parameters (VO_2_max (mL/min; mL/min/kg); oxygen pulse (mL), RER) were measured and the subjective feeling of exertion was recorded using the Borg scale [6].

To practically assess the feasibility of our SCT in clinical routines, we additionally enrolled five patients with Fontan circulation.

### 2.4. Statistical Analysis

The IBM SPSS (version 29.0.1 Statistics for Windows, IBM Corp., Armonk, NY, USA) program was utilized for statistical analysis. A descriptive analysis was conducted and reported as means ± standard deviations. Simple correlations were performed using the Spearman rho and Pearson correlation coefficients, depending on whether the correlated variables were normally distributed or not. The significance level was set at *p* < 0.05.

## 3. Results

In our study, we included 28 healthy children and young adults (12 male and 16 female) aged 13.89 years (±2.63 years) with a BMI of 18.9 kg/m^2^ (±2.34 kg/m^2^), as detailed in Table 1. These participants achieved a mean 6MWD of 753.0 m (±74.1 m) in the 6MWT, with a mean tSCT of 50.2s (±5.57 s) and a mean SCT-Index of 14.1 (±4.88). Additionally, the subjects attained a mean VO_2_max of 2573.9 mL/min (±983.61 mL/min) and a mean VO_2_max/kg of 48.3 mL/min/kg (±8.2 mL/min/kg). Maximal O_2_ pulse was measured at 13.1 mL (±5.83 mL). (Table 2).

**Figure 1:**
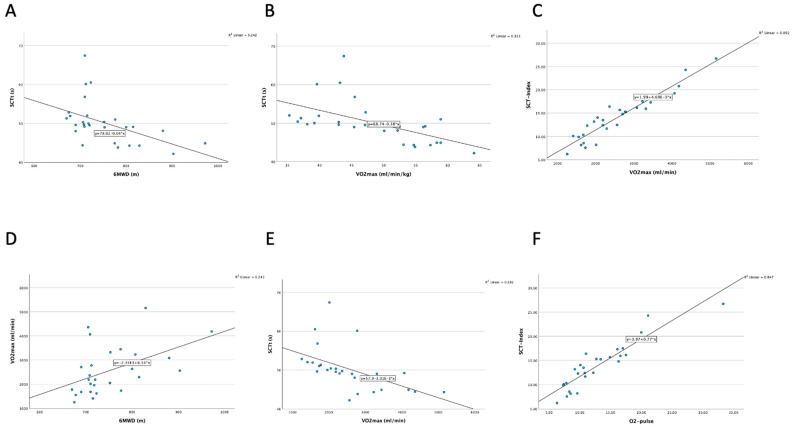
(**A**) Moderate correlation of 6MWD to tSCT (r = −0.544; *p* = 0.003). (**B**) Strong correlation of tSCT to VO_2_max (mL/min/kg) (r = −0.672, *p* < 0.001. (**C**) Very strong correlation of SCT-Index to VO_2_max (mL/min) (r = 0.927; *p* ≤ 0.001). (**D**) Moderate correlation of 6MWD to VO_2_max (mL/min) (r = 0.473, *p* = 0.011). (**E**) Strong correlation of tSCT to VO_2_max (mL/min) (r = −0.764; *p* ≤ 0.001). (**F**) Very strong correlation of oxygen pulse to SCT-Index (r = 0.921, *p* ≤ 0.001).

A very strong correlation was observed between SCT-Index and O_2_ pulse (r = 0.921; *p* < 0.001). In addition, when comparing the time achieved during SCT (tSCT) with VO_2_max/kg (mL/min/kg) and VO_2_max (mL/min), strong correlations were found (r = −0.672; *p* < 0.001 and r = −0.764; *p* < 0.001) (see Figure 1). Finally, we determined a very strong correlation between SCT-Index and VO_2_max (mL/min) (r = 0.927; *p* < 0.001) and a moderate correlation between SCT-Index and VO_2_max/kg (mL/min/kg) (r = 0.407; *p* = 0.054). There was a moderate correlation between the distance walked during the 6MWT and VO_2_max/kg (mL/min/kg) (r = 0.569; *p* = 0.002) and only a moderate correlation with VO_2_max (mL/min) (r = 0.473; *p* = 0.011). When comparing the SCT-Index with the 6MWD, there was a weak correlation (r = 0.351; *p* = 0.067).

For the Fontan patients, we found a very strong correlation between the SCT-Index and VO_2_max (mL/min) (r = 0.929; *p* = 0.022) and between the SCT-Index and maximum O_2_ pulse (r = 0.928; *p* = 0.023).

## 4. Discussion

CPET has various indications and it is still not universally accessible as it is an expensive examination requiring a lot of equipment and time.

Our study was a pilot study with the aim to define a simple Stair Climbing Test (SCT) for the assessment of the fitness of healthy children and adolescents comparable to CPET and the 6MWT. A previous study by our working group had already shown a significant correlation between the SCT using our standardized protocol and the parameters measured in spiroergometry in healthy and obese adults [20]. In this study, we also tested this assumption for healthy 10–18-year-old children and adolescents. We also performed the SCT in five patients with complex congenital heart disease and a priori reduced exercise performance to evaluate whether it can also be used in individuals with CHD.

The gold standard for assessing aerobic fitness is VO_2_max and VO_2_peak, measured by CPET [2,21]. It can be challenging to reach a plateau in VO_2_ during exercise tests, especially for untrained subjects unfamiliar with the discomfort of strenuous activities. Therefore, in many cases, only VO_2_peak is attained, serving as a substitute for VO_2_max [11], while other criteria such as RER, heart rate, and the Borg scale are used to determine VO_2_max [16]. The VO_2_max reported in our results corresponds to the maximum amount of oxygen absorbed by the test subjects at the maximum load attained. As previously mentioned, the subjects had to achieve an RER of >1.0. RER objectively describes an individual’s level of exertion.

O_2_ pulse is the amount of oxygen absorbed per heartbeat and is calculated as follows: oxygen pulse = VO_2_ (mL/min)/heart rate [22,23]. Since O_2_ pulse is dependent on VO_2_, it is understandable that both VO_2_max and O_2_ pulse correlate very well with the SCT-Index, with only a minimal difference in the correlation.

In adults, other variations of the Stair Climbing Test have primarily been utilized in patients with lung diseases [18,24], orthopedic conditions, or neuromuscular impairment, each using different protocols and SCT procedures. These tests may serve as a predictive tool for postoperative complications and aid in identifying patients who could potentially benefit from an extra CPET [25]. In the orthopedic setting, the SCT offers valuable information to assess mobility in general and an individual’s capability to ascend and descend stairs, as well as evaluate lower limb strength [26]. Zaino et al. used an SCT including 14 steps to examine functional mobility in 47 children, 20 of them with cerebral palsy [27]. Regarding the level of exertion, which is needed to aim for a maximal exercise test, the needed vertical height seems to be 12meters or more. Therefore, our protocol includes four flights of stairs with a total vertical height over 12 m. Devendra et. al. also used a protocol including four flights of stairs to assess desaturation during exercise in patients with patent foramen ovale [28]. In contrast, in pediatrics, the 4 Stairs Climb Test (4SC) is used for the assessment of motion capability. This pediatric test is primarily intended for sick children and involves ascending and descending only over a height of 4 steps; therefore, it is hardly comparable to our study. It has demonstrated particular success in children and is widely employed for evaluating the motor function of patients with neuromuscular diseases [20,29]. It is essential to note that the 4SC test, although also assessing stair climbing, differs from the adult-focused SCT.

To the best of our knowledge, there are no comparable data for an SCT or SCT-Index in the literature for healthy 10–18-year-old children and adolescents that would be comparable to the test we present here. Nevertheless, there is a relevant approach for adults that aligns with our methodology and lends support to the results we presented. Cataneo et al. [30] conducted a study wherein they reported a moderate correlation (r = 0.515; *p* < 0.005) in their variation of the SCT-Index (called $\mathrm{power}\;=\;\frac{\mathrm{weight}\;\times \;\mathrm{height}\;\times \;\mathrm{gravity}}{\mathrm{time}}$) and a strong correlation (r = −0.707; *p* < 0.005) in tSCT with VO_2_max/KG. These results strengthen our general assumption and findings.

Our test protocol and measurements also revealed a strong correlation between the SCT-Index and VO_2_max. However, when considering VO_2_max/kg, the correlation was moderate. This could be due to the fact that physical performance, especially in children and adolescents, is complex and cannot simply be related to weight [21]. Blais et al. [21] therefore even suggest abandoning the indexing of VO_2_max based on weight. Instead, they advocate for more precise adjustments based on body size, emphasizing the importance of such adjustments to attain more accurate and meaningful results. However, for the sake of simplicity, we included only body weight as a factor in our SCT-Index as the body weight had to be lifted across the four floors. Since the subjects we included neither had a pre-existing disease nor were obese, the results should not be over- or underestimated.

The 6MWT is a submaximal test and is primarily used to evaluate functional status and monitor the response to medical interventions in individuals struggling with impaired exercise capacity and moderate to severe heart and lung diseases [17,31]. Additional unique challenges when using it with children include a potential lack of motivation and understanding regarding the necessity of the 6MWT [32]. This highlights the importance of considering age-specific factors in the interpretation of results.

In our study, we identified a moderate correlation between 6MWD and VO_2_max. These findings are in line with the results of two other studies. Specifically, Jalil et al. observed a strong correlation between the 6MWT and VO_2_max (*p* < 0.01, r = 0.723) in their research involving 8–17-year-old boys. Additionally, they reported similar outcomes from other studies encompassing sick children and adolescents, with correlations ranging from r = 0.34 to r = 0.76 [33]. Furthermore, Li et al. conducted a study focusing on 12–16 year old children and adolescents, and their results also indicated a moderate correlation between 6MWD and VO_2_max (r = 0.44, *p* ≤ 0.0001) [34]. The consistency across these studies reinforces the association between the distance covered in the 6 Min Walking Test and maximal oxygen uptake, mainly in healthy children.

Furthermore, we observed a weak correlation between the SCT Index and the 6MWD in our results. This may be explained by the fact that the SCT is a maximal exercise test and should be considered either complementary to or as a potential replacement for a maximal exercise test, e.g., spiroergometry, in some fields, or as a screening tool in institutions or countries without access to spiroergometry.

The excellent clinical correlation of the SCT in healthy children and adolescents was replicable in patients with univentricular hearts and Fontan physiology. We therefore aim to use this test in a larger population with congenital heart disease.

### Limitations

As our study is a pilot study, the data naturally only relate to a small cohort and may need confirmation on a larger scale and in different patient groups, for instance, subjects with congenital heart disease—in moderate cases maybe even before and after surgery—and children of a younger age. As all tests were performed on the same day, subjects potentially experienced exhaustion toward the end of the test period. However, we ensured that the subjects had adequate rest periods between tests and could recover effectively. We waited until the heart rate had returned to the baseline value, and the subjects subjectively reported feeling recovered before proceeding with each test. Treadmill spiroergometry was intentionally scheduled as the final test, as subjects were expected to put maximal effort into it.

It is important to note that the performance of children and adolescents may be highly dependent on their motivation. To attenuate this, we ensured consistent motivation and encouragement for all test subjects, employing identical sentences and wording for both the stair climbing test and spiroergometry. Furthermore, it is worth mentioning that the expressions to be used with the 6MWT are fixed anyways. Additionally, a specific minimum load was attained during spiroergometry by reaching the prescribed minimum RER.

## 5. Conclusions

Physical activity, especially high cardiorespiratory fitness (CRF), offers essential health benefits for children and adolescents. We were able to show that there is a significant correlation between our newly developed and standardized SCT and a standardized treadmill CPET in healthy children as well as in patients with Fontan circulation. Consequently, the SCT can serve as a valuable supplement to CPET and, in specific contexts, even as a substitution for treadmill CPET, especially when the primary goal is assessing fitness or exercise capacity rather than detecting cardiorespiratory diseases. This underscores the SCT’s advantages as a simple, quick, affordable, and reliable standardized exercise test for routine CRF assessment in healthy children, with potential applications in patients with congenital heart disease.

## Figures and Tables

**Table 1:** Descriptive statistics of the cohort (showing means and standard deviations). N = number.

	All Healthy	Male	Female	Fontan
N	28	12	16	5
Age [years]	13.89 (±2.63)	14.08 (±2.43)	13.75 (±2.84)	13.40 (±3.29)
BMI [kg/m^2^]	18.9 (±2.34)	19.26 (± 3.03)	18.63 (±1.73)	18.51 (±3.29)
Height [m]	1.64 (±0.15)	1.70 (± 0.20)	1.61 (±0.09)	1.58 (±0.23)
Weight [kg]	52.53 (±15.17)	57.83 (± 20.22)	48.56 (±8.66)	47.8 (±18.67)

**Table 2:** Descriptive statistics of the exercise test (showing means and standard deviations).

	All Healthy	Male	Female	Fontan
6MWD [m]	752.96 (±74.1)	783.83 (±86.62)	729.81 (±55.19)	493.2 (±103.35)
VO_2_max [mL/min]	2573.93 (±983.61)	3160.42 (±1146.61)	2134.06 (±543.89)	1562.8 8 (±741.85)
VO_2_max/kg [mL/min/kg]	48.28 (±8.2)	54.79 (±6.33)	43.39 (±5.67)	32.08 (±5.41)
Oxygen pulse [mL]	13.09 (±5.83)	15.88 (±7.23)	11.0 (±3.46)	9.16 (±4.38)
Respiratory quotient	1.15 (±0.07)	1.16 (±0.06)	1.15 (±0.09)	1.10 (±0.09)
tSCT [s]	50.16 (±5.57)	47.98 (±5.04)	51.8 (±5.53)	81.04 (±38.05)
SCT index	14.06 (±4.88)	16.05 (±6.16)	12.56 (±3.08)	9.99 (±6.73)

## Data Availability

The data presented in this study are available in article.
